# No detectable impact of short-term treatment delays on lung cancer survival

**DOI:** 10.1007/s11357-025-01684-9

**Published:** 2025-05-07

**Authors:** Zoltan Ungvari, Mónika Fekete, Annamaria Buda, Andrea Lehoczki, János Tibor Fekete, Gyöngyi Munkácsy, Péter Varga, Anna Ungvari, Balázs Győrffy

**Affiliations:** 1 Vascular Cognitive Impairment, Neurodegeneration and Healthy Brain Aging Program, Department of Neurosurgery, University of Oklahoma Health Sciences Center, Oklahoma City, OK USA; 2 Stephenson Cancer Center, University of Oklahoma, Oklahoma City, OK USA; 3 Oklahoma Center for Geroscience and Healthy Brain Aging, University of Oklahoma Health Sciences Center, Oklahoma City, OK USA; 4 Department of Health Promotion Sciences, College of Public Health, University of Oklahoma Health Sciences Center, Oklahoma City, OK USA; 5 International Training Program in Geroscience, Doctoral College, Health Sciences Division/Institute of Preventive Medicine and Public Health, Semmelweis University, Budapest, Hungary; 6 Institute of Preventive Medicine and Public Health, Semmelweis University, Semmelweis University, Budapest, Hungary; 7 Jozsef Fodor Center for Prevention and Healthy Aging, Semmelweis University, Budapest, Hungary; 8 Department of Public Health and Epidemiology, Faculty of Medicine, HUN-REN-DE Public Health Research Group, University of Debrecen, 4012 Debrecen, Hungary; 9 Doctoral College, Health Sciences Division, Semmelweis University, Budapest, Hungary; 10 Dept. of Bioinformatics, Semmelweis University, 1094 Budapest, Hungary; 11 Cancer Biomarker Research Group, Institute of Molecular Life Sciences, HUN-REN Research Centre for Natural Sciences, 1117 Budapest, Hungary; 12 Dept. of Biophysics, Medical School, University of Pecs, 7624 Pecs, Hungary

**Keywords:** Mortality, Lung cancer, Clinical outcomes, Oncology, Treatment initiation, Hazard ratio, Cancer prognosis, Disease progression, Cohort study

## Abstract

Timely initiation of treatment is a core principle of oncologic care, especially for aggressive cancers such as lung cancer. However, the real-world impact of short-term delays in treatment initiation on survival outcomes in lung cancer remains unclear. This meta-analysis evaluates the association between treatment delays of 4, 8, and 12 weeks and all-cause mortality in lung cancer patients. A systematic search was conducted in PubMed, Scopus, and Web of Science for studies published between 2000 and 2025. Of 5360 screened records, 15 studies were included, comprising 16 cohorts for overall survival of lung cancer patients. Hazard ratios (HRs) for 4-, 8-, and 12-week treatment delays were estimated using random-effects meta-analyses. Heterogeneity was measured with the *I*^2^ statistic, and publication bias was assessed using funnel plots and Egger’s test. No significant association was found between treatment delay and survival at any of the time points. Pooled HRs were 1.00 (95% CI, 0.99–1.02) for a 4-week delay, 1.01 (95% CI, 0.99–1.03) for an 8-week delay, and 1.01 (95% CI, 0.98–1.05) for a 12-week delay. Despite high heterogeneity (*I*^2^ = 97%), no evidence of publication bias was detected. This meta-analysis found no significant impact of short-term treatment delays (up to 12 weeks) on mortality in lung cancer patients. These findings challenge the assumption that brief delays universally worsen outcomes and underscore the importance of individualized treatment planning and prioritization.

## Introduction

Timely initiation of treatment is considered a critical determinant of survival in many cancers [1, 2], particularly in aggressive malignancies such as lung cancer. Delays in care can occur at various stages—from diagnosis and staging to treatment planning and initiation—and may stem from patient-related, provider-level, or systemic factors [3–10]. It is commonly assumed that even short delays can permit tumor progression and lead to poorer outcomes. The COVID-19 pandemic has particularly underscored the potential harm of treatment delays [10, 11].

Indeed, prior studies in breast [12–32] and colorectal cancers [20, 24, 33–50] have shown that each 4-week delay in initiating treatment is associated with a measurable increase in mortality. However, the impact of short-term delays in lung cancer remains less clear [8]. Lung cancer is biologically heterogeneous, with varying growth rates, disease trajectories, and responses to treatment depending on histologic subtype, stage at diagnosis, and molecular characteristics. This heterogeneity may contribute to inconsistent findings in the literature regarding treatment delays and survival outcomes [8].

Moreover, clinical urgency in lung cancer often leads to expedited care, especially in symptomatic patients with advanced disease [51]. In such contexts, delays may reflect appropriate diagnostic workup, medical optimization, or triage prioritization—rather than system inefficiencies. Thus, the assumption that short-term delays are uniformly harmful in lung cancer may not be supported by empirical data.

To clarify this issue, we conducted a systematic review and meta-analysis of published studies examining the association between treatment delays of 4, 8, and 12 weeks and overall survival in patients with lung cancer. By synthesizing data from over 16 cohorts, we aimed to provide evidence-based insight into whether treatment initiation timing significantly affects lung cancer prognosis.

## Methods

### Literature search

We conducted this meta-analysis to explore the association between delayed initiation of therapy and mortality in lung malignancies. Our investigation encompassed various treatment approaches, including surgical resection, systemic anticancer therapy, and radiation treatment, as well as their temporal classifications, such as preoperative neoadjuvant and postoperative adjuvant interventions.

To identify relevant studies, we performed a systematic literature search across three major biomedical databases: PubMed, Scopus, and Web of Science. We focused on publications from 2000 to 2025, using the following key terms: “lung cancer” AND “treatment delay” AND “mortality OR survival.”

### Filtering and data extraction

To ensure the inclusion of high-quality studies, we established predefined eligibility criteria. We selected prospective and retrospective cohort studies that assessed the impact of treatment delays on mortality in lung cancer patients. Studies were required to report hazard ratios (HR) quantifying the relationship between delayed treatment initiation and overall survival, with a minimum follow-up period of 30 days. We excluded animal studies, in vitro experiments, and theoretical models, as well as non-English publications and studies with methodological limitations that could compromise validity or included ineligible patient populations.

Data extraction was carried out independently by two researchers who systematically collected study-specific details, such as authorship, publication year, and geographical origin. We also recorded information on the cancer subtype, the type of treatment administered, the reported HRs evaluating the impact of treatment postponement on survival outcomes, and the duration and extent of treatment delays. In cases where discrepancies arose, we resolved them through consensus-based discussions between the two researchers.

### Statistical methods

We applied two statistical approaches to estimate the hazard ratio (HR) as the primary outcome variable in our meta-analysis of lung cancer studies. When studies did not specify a reference period, we standardized reported HR or odds ratio (OR) values using the following formula: HR per *X*-month delay = (HR per 4-week delay)^(*X* weeks delay/4 weeks delay) [1]. If a defined reference time was available, we utilized a weighted linear regression model to assess the association between treatment delay (in weeks) and the log-transformed HR for patient survival. Hazard ratio estimates with 95% confidence intervals (CIs) were calculated for delays of 4, 8, and 12 weeks.

To generate pooled risk estimates, we employed a random-effects model, which accounts for variability across studies and improves the generalizability of the findings. Forest plots were constructed to visually represent individual study results along with the overall summary estimate, facilitating interpretation and identifying potential heterogeneity between studies. All statistical analyses were conducted using the online platform MetaAnalysisOnline.com [52].

#### Analysis of variability and publication bias

To assess variability among studies, we applied Cochran’s *Q* test and the *I*^2^ statistic. The *Q* test, based on a chi-squared distribution, determined whether observed differences in effect sizes exceeded those expected by chance, while the *I*^2^ statistic quantified the proportion of total variance attributable to actual study differences rather than random fluctuations.

To examine potential publication bias, we generated funnel plots to illustrate the relationship between study effect sizes and their precision. Asymmetry in these plots suggested possible bias. Additionally, Egger’s regression analysis was conducted to statistically evaluate the correlation between effect sizes and their standard errors, providing a quantitative measure of publication bias.

## Results

### Included studies

**Fig. 1:**
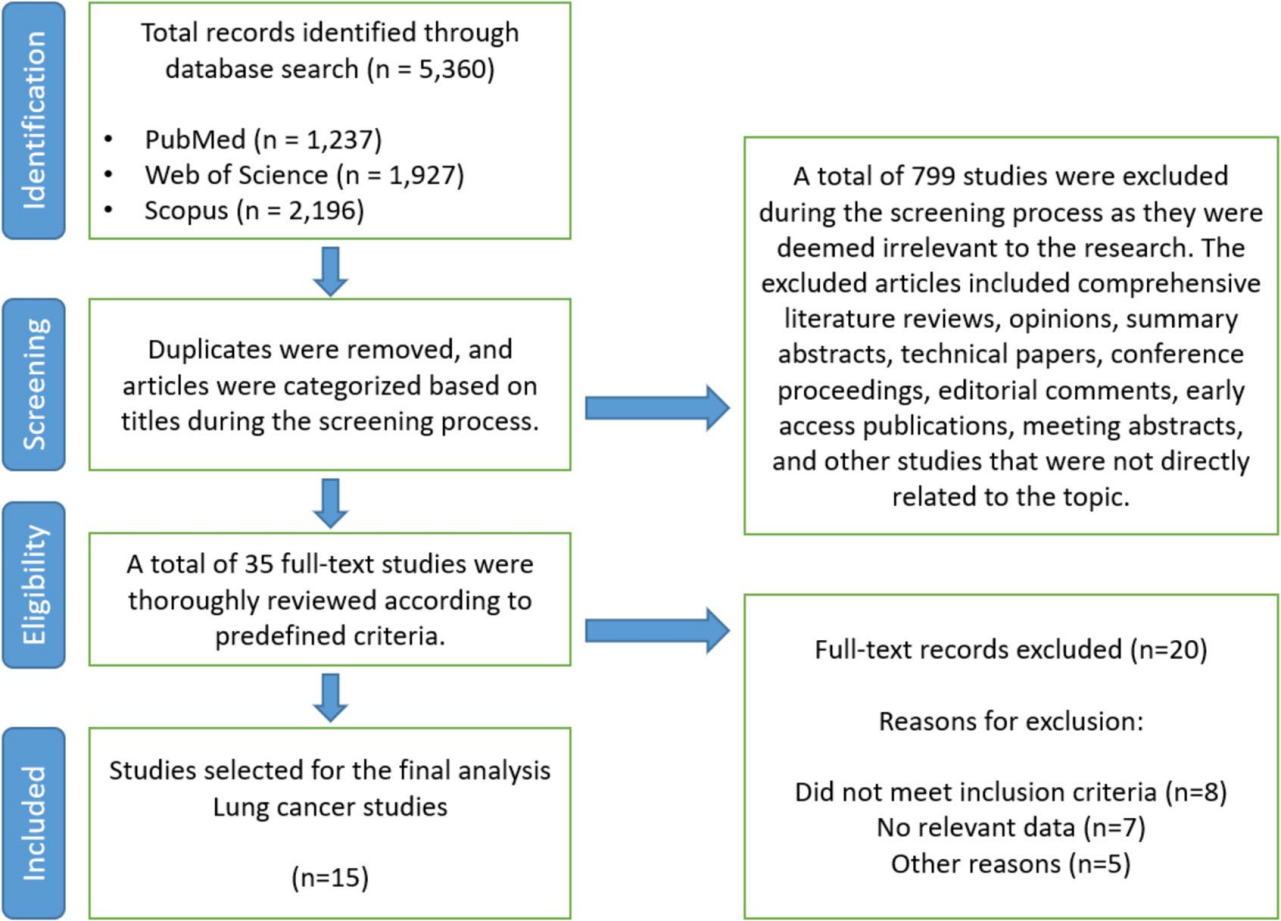
Flow diagram illustrating the study selection process

A systematic search of PubMed, Web of Science, and Scopus identified 5360 potentially relevant records (Fig. 1). During the screening process, 799 studies were excluded based on predefined criteria, including literature reviews, case reports, guidelines, expert opinions, summary abstracts, and studies unrelated to the research topic. After removing duplicates and performing a title-based screening, 35 articles remained. Of these, 20 were excluded due to not meeting inclusion criteria (*n* = 8), lack of relevant data (*n* = 7), or other reasons (*n* = 5). Ultimately, 15 studies focusing on lung cancer were included in the final analysis [24, 53–66].

The analysis incorporated a uniform dataset across all delay intervals, including 16 cohorts evaluating all-cause mortality. A detailed summary of studies assessing the impact of treatment delay on survival outcomes of lung cancer patients is provided in Table 1.

**Table 1:** Overview of studies evaluating the association between treatment delays and survival outcomes in lung cancer patients. The table summarizes key data from individual studies, including the author and year of publication. Hazard ratios (HR) or odds ratios (OR) are reported for treatment delays of 4, 8, and 12 weeks, with corresponding confidence intervals (CI). Additionally, the table details the total sample size and the number of cases analyzed in each study. Abbreviations:* CI*, confidence interval; *HR*, hazard ratio; *OR*, odds ratio

Author	Year	4-week delay			8-week delay			12-week delay			HR/OR	Total *n*	Case *n*
		**Rate**	**95% CI**		**Rate**	**95% CI**		**Rate**	**95% CI**				
Abrao et al	2018	0.768	0.678	0.877	0.590	0.460	0.770	0.453	0.312	0.676	HR	359	278
Booth et al	2013	1.000	0.996	1.000	1.000	0.992	1.000	1.000	0.988	1.000	OR	1032	16
Cushman et al	2021	1.044	1.040	1.049	1.091	1.081	1.101	1.139	1.123	1.156	HR	140,455	9809
Diaconescu et al	2011	0.972	0.952	0.992	0.946	0.906	0.985	0.920	0.863	0.977	HR	495	252
Gomez et al. (localized disease)	2015	0.886	0.837	0.927	0.786	0.700	0.860	0.696	0.585	0.797	OR	7960	3069
Gomez et al. (regional disease)	2015	1.040	0.992	1.087	1.081	0.984	1.182	1.124	0.976	1.285	OR	8962	4158
Gonzalez-Barcala et al	2013	0.675	0.573	0.796	0.456	0.329	0.633	0.308	0.188	0.504	HR	307	154
Heiden et al	2021	1.042	1.020	1.063	1.085	1.040	1.129	1.130	1.060	1.200	HR	9904	203
Kanarek et al	2014	1.026	1.000	1.059	1.054	1.000	1.122	1.082	1.000	1.188	HR	174	56
Myrdal et al	2004	0.969	0.957	0.983	0.940	0.915	0.965	0.911	0.875	0.949	HR	466	322
Salazar et al	2017	1.018	0.979	1.058	1.036	0.959	1.119	1.054	0.940	1.183	HR	12,473	1147
Samson et al	2015	1.002	1.001	1.003	1.004	1.002	1.007	1.006	1.003	1.011	HR	27,022	713
Sheinson et al	2020	2.604	1.177	5.743	6.782	1.385	32.986	17.661	1.630	189.450	HR	185	159
Szejniuk et al	2021	0.766	0.543	1.098	0.586	0.295	1.205	0.449	0.160	1.323	HR	166	45
Tsai et al	2020	1.039	1.015	1.064	1.079	1.030	1.131	1.121	1.045	1.203	HR	42,962	35,431
Yun et al	2012	1.160	1.060	1.270	1.346	1.124	1.613	1.561	1.191	2.048	HR	9094	4538

### Effect of a 4-week delay in treatment

**Fig. 2:**
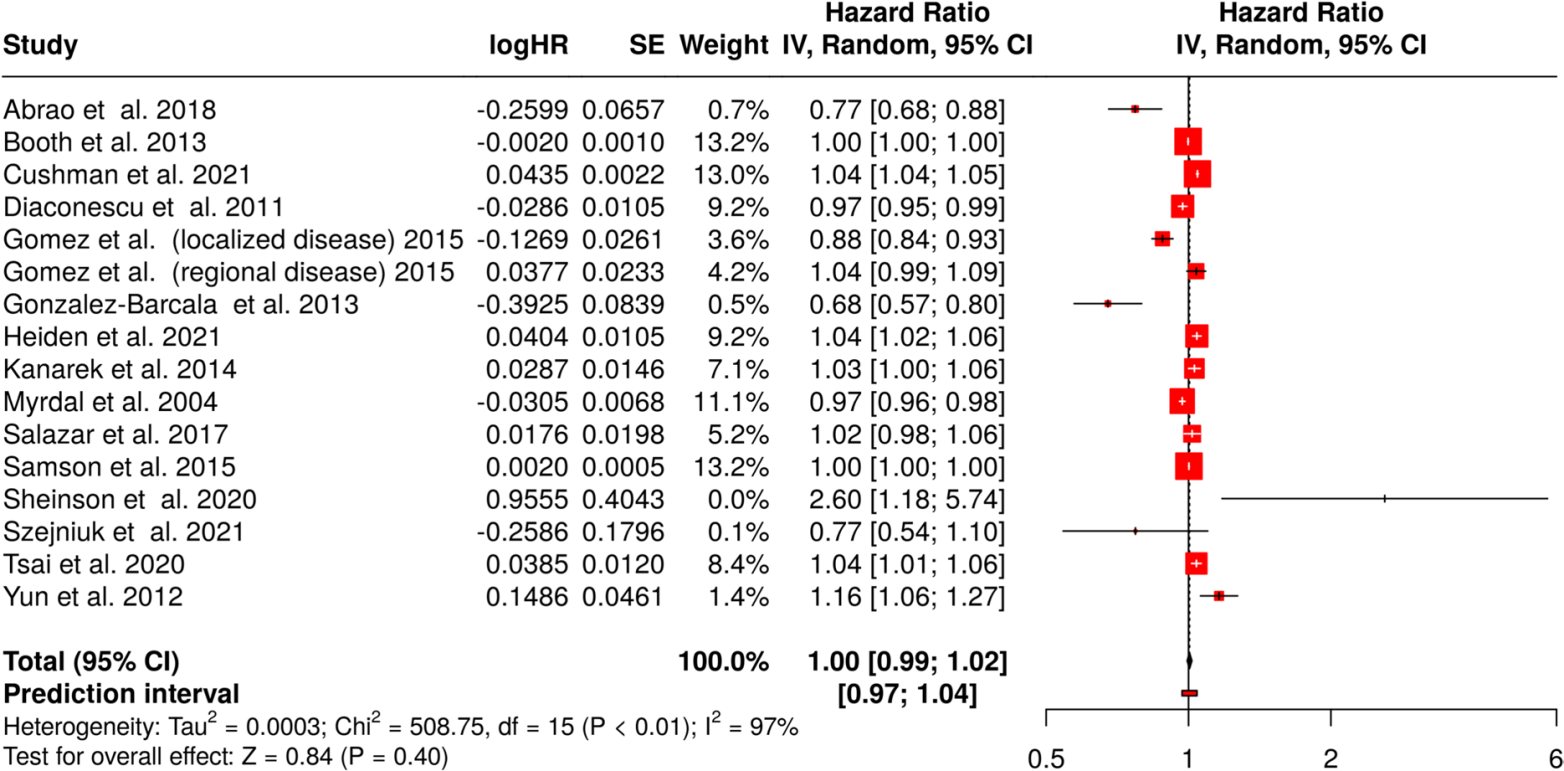
Meta-analysis of the association between a 4-week treatment delay and all-cause mortality in lung cancer patients. The forest plot presents hazard ratios (HRs) with 95% confidence intervals (CIs) for individual studies and the pooled estimate. The overall HR was 1.00 (95% CI, 0.99–1.02), indicating no significant association between treatment delay and mortality (*Z* = 0.84, *p* = 0.40). The prediction interval ranged from 0.97 to 1.04. Heterogeneity was high (*I*^2^ = 97%). The diamond represents the pooled effect size. Abbreviations: CI, confidence interval; HR, hazard ratio; IV, inverse variance; SE, standard error

Using a random-effects model with inverse variance method to estimate the hazard ratio (HR), the pooled HR was 1.00 (95% CI, 0.99–1.02), indicating no statistically significant effect of 4-week delay in treatment on survival of lung cancer patients (Fig. 2). The test for overall effect did not reveal a significant effect. However, substantial heterogeneity was observed (*p* < 0.01), suggesting considerable variation in effect sizes across studies. The *I*^2^ statistic demonstrated that 97% of the observed variability was attributable to heterogeneity rather than random variation.

**Fig. 3:**
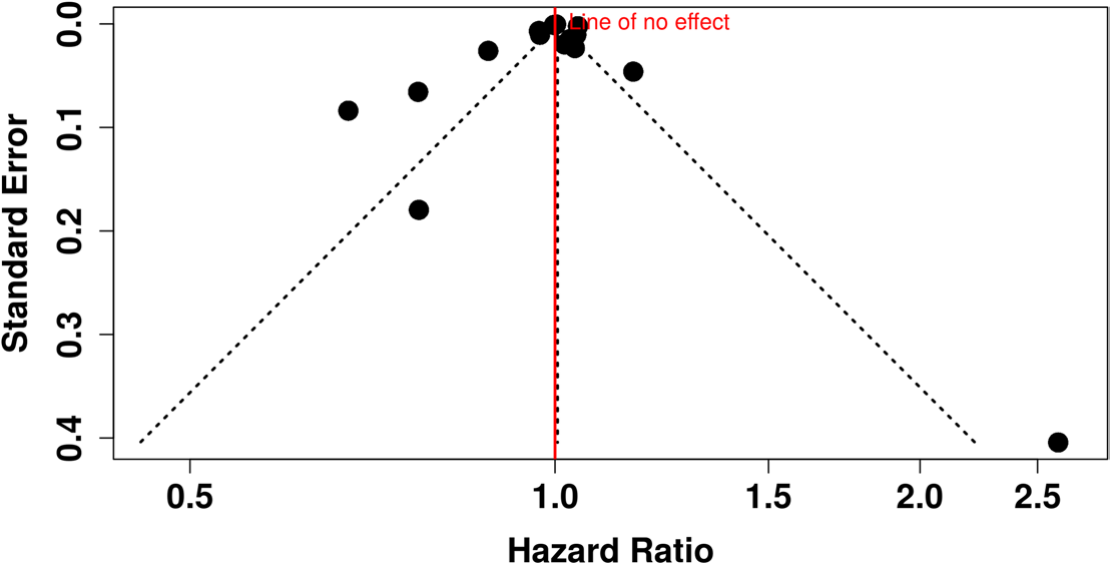
Funnel plot assessing publication bias in studies on the effect of treatment delay on lung cancer survival

Assessment of publication bias through a funnel plot did not suggest asymmetry (Fig. 3). Additionally, Egger’s test provided no evidence of bias (intercept, 0.40; 95% CI, − 2.91 to 3.7; *t* = 0.236; *p* = 0.817), reinforcing the robustness of the findings.

### Effect of an 8-week delay in treatment

**Fig. 4:**
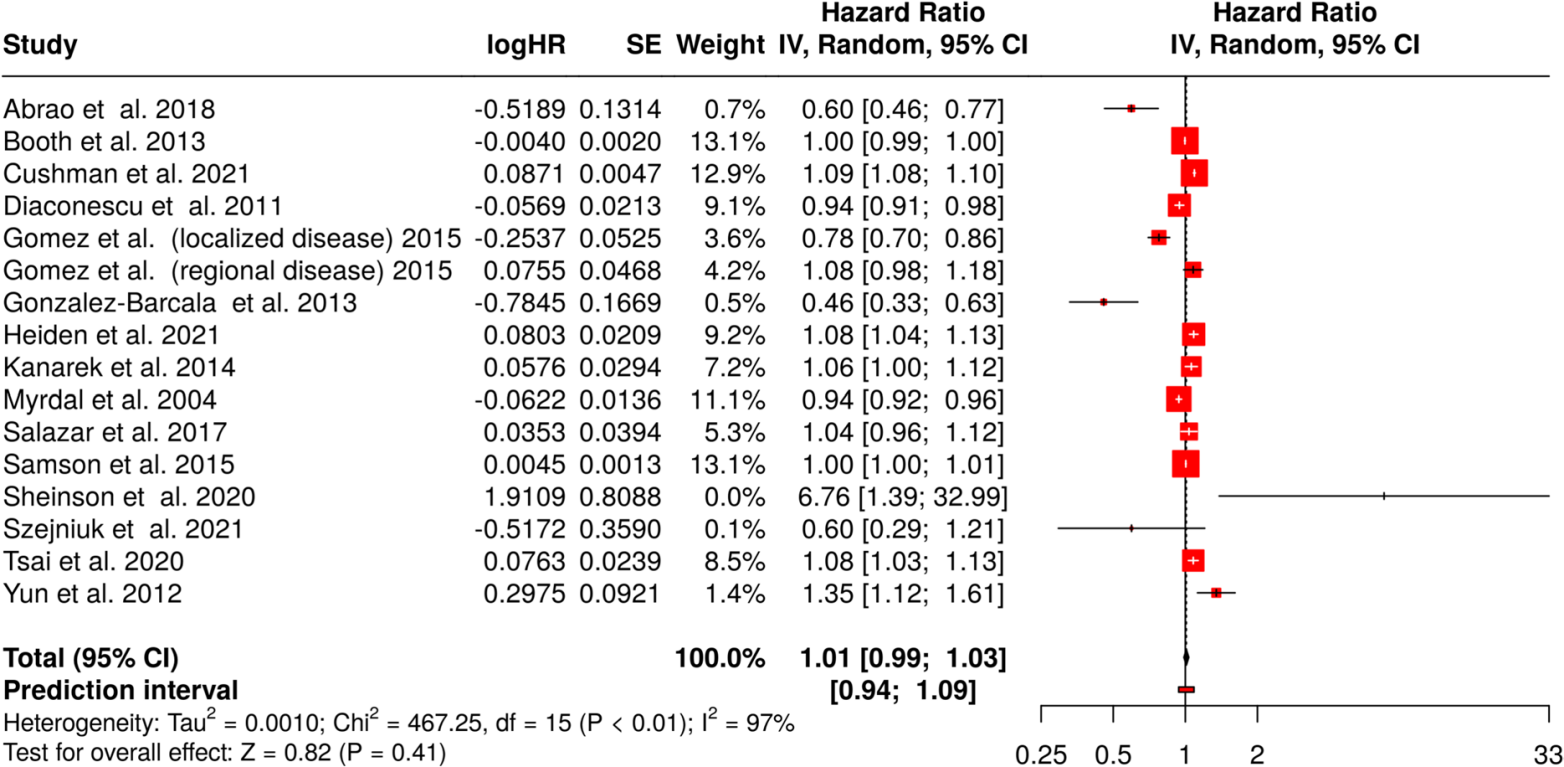
Forest plot depicting the impact of an 8-week treatment delay on all-cause mortality in lung cancer patients. The combined HR of 1.01 (95% CI, 0.99–1.03) indicates no significant effect of treatment delay on mortality (*Z* = 0.82, *p* = 0.41). High heterogeneity (*I*^2^ = 97%) suggests variability among studies. Abbreviations: CI, confidence interval; HR, hazard ratio; IV, inverse variance; SE, standard error

A meta-analysis incorporating the same 16 cohorts was conducted (Fig. 4) when investigating the effect of 8-week delay in treatment. The pooled HR was 1.01 (95% CI, 0.99–1.03), again showing no statistically significant effect. The test for overall effect remained nonsignificant. Notably, heterogeneity was substantial (*p* < 0.01), reflecting discrepancies in effect magnitude and/or direction across studies. The *I*^2^ statistic indicated that 97% of the variation stemmed from true heterogeneity rather than random chance.

Examination of potential publication bias did not reveal substantial asymmetry in the funnel plot (*data not shown*). This was further supported by Egger’s test, which failed to detect significant deviation from symmetry (intercept, 0.31; 95% CI, − 2.91 to 3.53; *t* = 0.189; *p* = 0.853).

### Effect of a 12-week delay in treatment

**Fig. 5:**
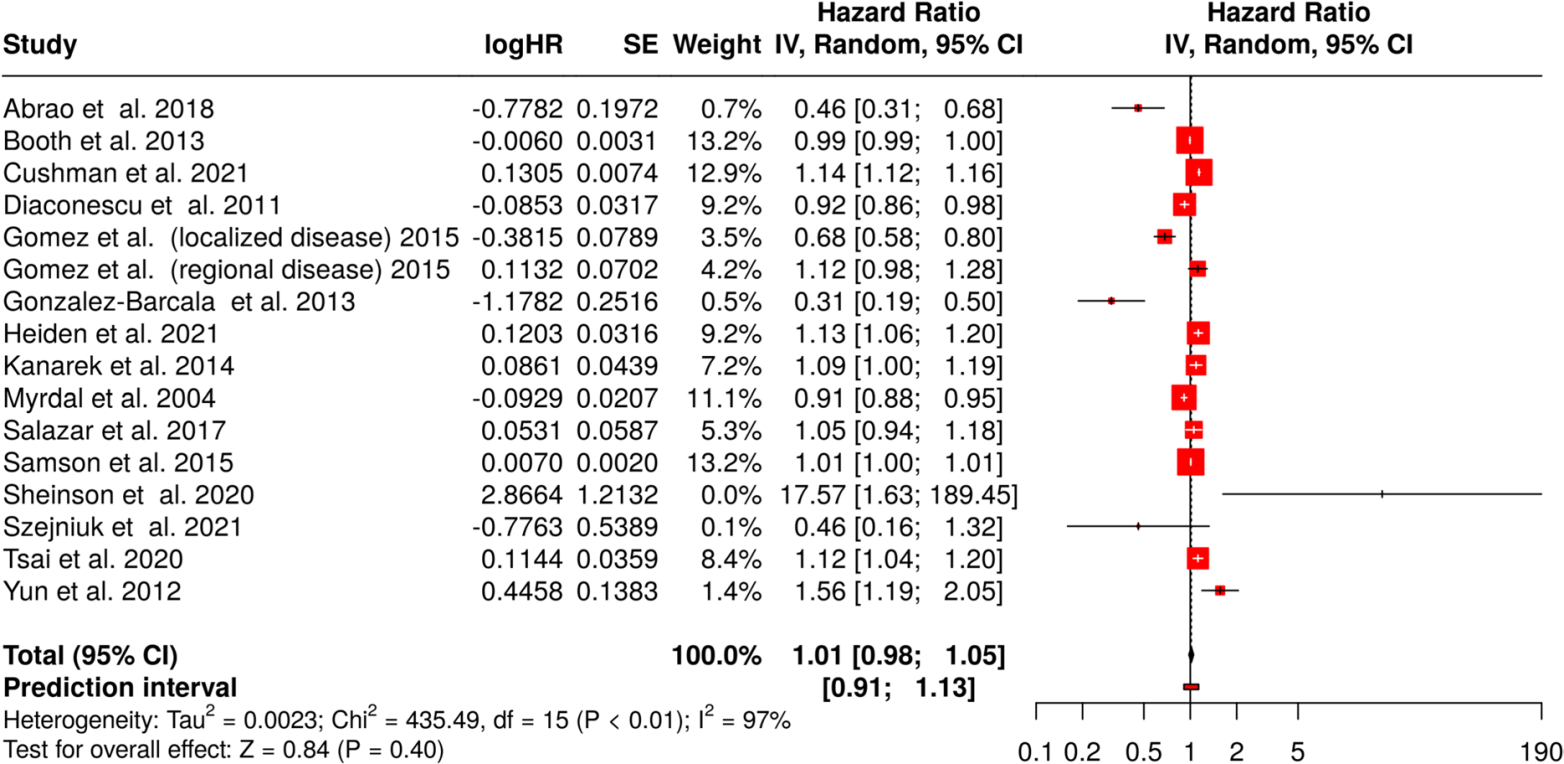
The impact of a 12-week treatment delay on all-cause mortality in lung cancer patients. The pooled HR of 1.01 (95% CI, 0.98–1.05) indicates no statistically significant effect on survival. Considerable heterogeneity is observed (*I*^2^ = 97%), suggesting substantial differences between studies. Abbreviations: CI, confidence interval; HR, hazard ratio; IV, inverse variance; SE, standard error

The impact of a 12-week delay was assessed using the same methodological framework. The meta-analysis yielded a pooled HR of 1.01 (95% CI, 0.98–1.05), as shown in Fig. 5, indicating no significant association between delayed treatment and increased mortality risk. The overall effect test did not reach statistical significance. Considerable heterogeneity was again detected (*p* < 0.01), highlighting variation in effect size across studies. The *I*^2^ value of 97% confirmed that nearly all observed differences originated from heterogeneity rather than random error.

Evaluation of publication bias did not indicate notable funnel plot asymmetry (*data not shown*). Similarly, Egger’s test ruled out substantial bias (intercept, 0.28; 95% CI, − 2.83 to 3.40; *t* = 0.179; *p* = 0.861), reinforcing the reliability of the findings.

## Discussion

This meta-analysis of 16 cohorts found no statistically significant association between treatment delays of 4, 8, or 12 weeks and all-cause mortality in lung cancer patients. While this contrasts with findings in other cancers, such as breast [12–32] and colorectal malignancies [20, 24, 33–50], the results are consistent with a subset of previous lung cancer studies reporting limited or no impact of short-term delays on survival [67]. Importantly, our findings challenge the universal assumption that all treatment delays in lung cancer result in poorer outcomes.

Several factors may account for the apparent lack of effect. First, the clinical course of lung cancer is highly variable, and its natural history depends on multiple factors, including histologic subtype, molecular profile, and disease stage at presentation [68]. For example, indolent tumors such as certain adenocarcinomas may be less affected by short-term delays, while rapidly progressing small-cell or aggressive non-small cell lung cancers may present so acutely that delays are inherently less common or less tolerated [69]. As a result, studies may preferentially include patients whose disease biology allowed for treatment deferral, introducing selection bias. Second, the high heterogeneity observed across studies likely reflects both clinical and methodological variability. Differences in study design, patient populations, treatment modalities (e.g., surgery, chemotherapy, radiation), and definitions of delay (e.g., diagnosis-to-treatment vs. referral-to-treatment) complicate cross-study comparisons. Additionally, few studies reported whether delays were driven by patient-related versus system-related factors, limiting the interpretability of delay impact across different care settings.

Second, lung cancer care often necessitates thorough diagnostic and staging procedures—such as biopsy, PET/CT imaging, and pulmonary function testing—prior to determining treatment strategy [8, 70]. In this context, brief delays may reflect appropriate clinical decision-making rather than system inefficiency [51, 71]. Thus, some of the included delays may be clinically justified and not indicative of suboptimal care, potentially attenuating any survival signal [71]. In fact, a large retrospective study of 693,554 non-small cell lung cancer patients from the National Cancer Data Base found that time-to-treatment longer than 4 weeks was associated with a lower overall risk of death across all cancer stages, possibly reflecting the benefits of thorough pre-treatment evaluation [71]. Importantly, we were unable to conduct subgroup analyses by treatment modality (e.g., surgery vs. chemotherapy vs. radiation), as this information was not consistently available across studies. This remains an important limitation, as the consequences of delay may vary significantly by modality and treatment intent.

Despite the null findings, this analysis should not be interpreted as suggesting that treatment delays are inconsequential. Rather, it highlights the need for personalized, context-aware triage systems that prioritize patients based on tumor aggressiveness, clinical stability, and resource availability [71, 72]. Timely treatment remains a cornerstone of high-quality oncology, and triage strategies should continue to be guided by disease biology, symptom burden, and treatment intent [71, 73]. In clinical practice, decision-making about treatment urgency should consider the natural history of the tumor [51, 71]. For instance, in operable non-small cell lung cancer, where the disease is localized and curative resection is possible, minimizing surgical delays is crucial [51, 55, 71]. Delays beyond 4 to 12 weeks in this subgroup have been associated with upstaging and decreased resectability in some retrospective series [71]. Similarly, in patients receiving definitive chemoradiation for locally advanced disease, prolonged intervals between diagnosis and therapy initiation may allow for tumor progression or functional decline that precludes curative treatment. Conversely, for certain patients with metastatic disease or indolent tumors identified incidentally, a short delay may offer an opportunity for further diagnostic clarification, molecular testing, or optimization of performance status without negatively impacting prognosis. Therefore, lung cancer management requires a nuanced, risk-adapted approach to triage—balancing urgency with the need for comprehensive workup and individualized planning [71]. In settings with constrained capacity—such as during public health emergencies [72]—our results may provide some reassurance that modest, short-term delays in certain lung cancer patients may not compromise survival. Nonetheless, the possibility remains that specific subgroups—such as patients with high-grade tumors, limited-stage disease undergoing curative surgery, or those with targetable mutations—could still be negatively affected by delays. Our analysis was not powered to detect such subgroup effects, and further research is warranted to define high-risk populations for whom time-to-treatment is a critical determinant of outcome.

Several limitations should be acknowledged. First, this meta-analysis is based on observational data, which are inherently subject to confounding and bias. Although most included studies adjusted for basic demographic and clinical variables, unmeasured confounders—such as socioeconomic status, comorbidity burden, or access to care—may have influenced both treatment timing and outcomes. Second, we observed significant heterogeneity across studies in delay definitions, treatment modalities, and patient populations. For example, time to surgery may not be equivalent in impact to time to systemic therapy or radiation, and many studies did not distinguish between these modalities. Third, the analysis does not account for tumor subtype, molecular profile, or treatment intent (curative vs. palliative), which are critical to assessing the effect of delays. Fourth, our analysis focused on delays up to 12 weeks; the impact of longer delays remains uncertain and may be clinically relevant in real-world settings. Finally, the lack of detailed data on the source and context of delays—whether patient-driven, system-driven, or clinically warranted—limits our ability to interpret causality.

In conclusion, this meta-analysis found no significant association between short-term treatment delays and all-cause mortality in lung cancer, contrasting with evidence from other high-mortality cancers such as breast and colorectal malignancies. These findings challenge the blanket assumption that every delay is detrimental and highlight the need for a more nuanced, context-specific approach to lung cancer management. Rather than applying universal time-to-treatment benchmarks, clinical decision-making should be guided by tumor biology, disease stage, and patient condition. In early-stage, operable NSCLC, minimizing surgical delay remains essential to preserve curative potential, while in patients with indolent or advanced disease, a short delay may allow for essential diagnostics and multidisciplinary planning without compromising survival. To translate these insights into practice, we recommend prioritizing high-risk patients for expedited treatment, maintaining flexibility for others, investing in diagnostic efficiency, and monitoring institutional delay metrics. These findings may also inform national cancer control strategies, particularly during times of healthcare strain. Ultimately, while brief delays may not be harmful for many lung cancer patients, delivering timely, individualized care remains a cornerstone of quality oncology. Future research should aim to define which subgroups are most vulnerable to delay-related harm and to identify context-sensitive time-to-treatment thresholds that balance urgency with individualized care.
